# Current and Emerging Approaches to Study Microenvironmental Interactions and Drug Activity in Classical Hodgkin Lymphoma

**DOI:** 10.3390/cancers14102427

**Published:** 2022-05-14

**Authors:** Naike Casagrande, Cinzia Borghese, Donatella Aldinucci

**Affiliations:** Molecular Oncology Unit, Centro di Riferimento Oncologico di Aviano (CRO), IRCCS, 33081 Aviano, Italy; naike.casagrande@cro.it (N.C.); cpborghese@cro.it (C.B.)

**Keywords:** classical Hodgkin Lymphoma, tumor microenvironment, multiplex immunohistochemistry, heterospheroids, drug response testing, stroma-mediated drug resistance, immunosuppression, tumor education

## Abstract

**Simple Summary:**

In classical Hodgkin Lymphoma (cHL), the tumor microenvironment (TME) plays an important role in tumor progression and treatment response, making its evaluation critical for determining prognosis, treatment strategies and predicting an increase in drug toxicity. Therefore, there is a need to utilize more complex systems to study the cHL-TME and its interplay with tumor cells. To evaluate new anticancer drugs and to find the mechanisms of drug resistance, this review summarizes emerging approaches for the analysis of the TME composition and to identify the state of the disease; the in vitro techniques used to determine the mechanisms involved in the building of an immunosuppressive and protective TME; new 3-dimensional (3D) models, the heterospheroids (HS), developed to mimic TME interactions. Here, we describe the present and likely future clinical applications indicated by the results of these studies and propose a classification for the in vitro culture methods used to study TME interactions in cHL.

**Abstract:**

Classic Hodgkin lymphoma is characterized by a few tumor cells surrounded by a protective and immunosuppressive tumor microenvironment (TME) composed by a wide variety of noncancerous cells that are an active part of the disease. Therefore, new techniques to study the cHL-TME and new therapeutic strategies targeting specifically tumor cells, reactivating the antitumor immunity, counteracting the protective effects of the TME, were developed. Here, we describe new methods used to study the cell composition, the phenotype, and the spatial distribution of Hodgkin and Reed–Sternberg (HRS) cells and of noncancerous cells in tumor tissues. Moreover, we propose a classification, with increasing complexity, of the in vitro functional studies used to clarify the interactions leading not only to HRS cell survival, growth and drug resistance, but also to the immunosuppressive tumor education of monocytes, T lymphocytes and fibroblasts. This classification also includes new 3-dimensional (3D) models, obtained by cultivating HRS cells in extracellular matrix scaffolds or in sponge scaffolds, under non-adherent conditions with noncancerous cells to form heterospheroids (HS), implanted in developing chick eggs (ovo model). We report results obtained with these approaches and their applications in clinical setting.

## 1. Introduction

Classic Hodgkin lymphoma (cHL) is among the most common lymphoma subtypes in children and young adults in the Western world [1]. cHL is a highly curable disease (70–80%), even though long-term treatment toxicity, tumors refractory to treatments and the occurrence of relapse are unsolved problems [2]. Thus, the discovery of markers of response to conventional therapies, of new drugs and less toxic drug combinations remains a challenge [1].

**Figure 1:**
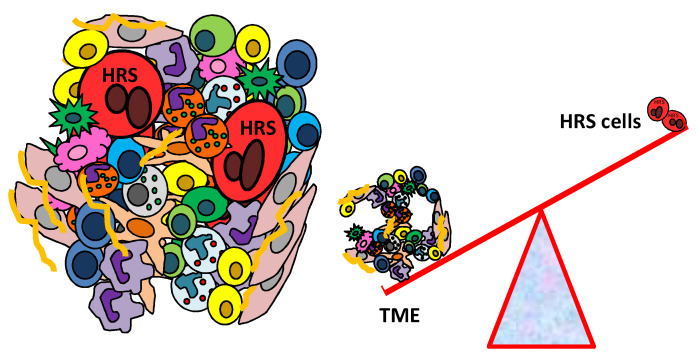
Schematic illustration of the cHL tumor microenvironment. cHL is characterized by a few tumor cells, called Hodgkin and Reed–Sternberg (HRS) cells, surrounded by an immune suppressive tumor microenvironment (TME) that includes T cells, eosinophils, tumor-associated macrophages (TAMs), a complex network of B cells, mast cells, plasma cells, fibroblasts, mesenchymal stromal cells (MSCs), endothelial cells and a rich extracellular matrix.

cHL comprises very few malignant cells, the so-called Hodgkin and Reed–Sternberg (HRS) cells. HRS cells show a similar gene expression pattern to CD30+ extrafollicular B cells, but typical B cell lineage markers are absent [3]. Tumor cells express high levels of CD30, CD40, IRF4, CD15 and constitutively active nuclear factor kappa B (NF-κB) [3]. HRS cells are embedded within an abundant immunosuppressive and protective tumor microenvironment (TME) [4], which includes T cells [5,6,7], eosinophils [8], tumor-associated macrophages (TAMs) [9], a complex network of B cells [10], mast cells [11,12], plasma cells [13], fibroblasts [14,15], mesenchymal stromal cells (MSCs) [16] and endothelial cells [17], as well as a rich extracellular matrix [18] (Figure 1).

By expressing immunosuppressive molecules and by secreting cytokines and extracellular vesicles (EVs), HRS cells can recruit and then “educate” noncancerous cells to become immunosuppressive/protective cells, such as tumor-associated macrophages (M2-TAMs), exhausted/anergic T-cells, and cancer-associated fibroblasts (CAFs) [19]. 

A recent definition of the spatial distribution of cHL identified a microenvironmental niche composed of programmed death ligand-1 (PD-L1)+ TAMs and PD-1+/CD4+ T cells that encircle PD-L1+ HRS cells [20]. In close contact with HRS cells, there are small anergic/exhausted CD4+ T cells (the so-called rosetting T cells) [5]. CD4+ T cells can directly interact with tumor cells and protect them from cytotoxic T cells and natural killer (NK) cells [5,6,21,22,23]. This compartmentalization of the cHL TME into a protective niche favors immune escape mechanisms, promotes tumor cell survival, and weakens the antitumor effects of chemotherapy and radiotherapy [3,23].

cHL is frequently related to the presence of Epstein–Barr virus (EBV) [24,25]. The percentage of EBV+cHL patients residing in low-resource nations is higher than in rich countries, such as those in Europe and North America [24,25]. Three proteins encoded by EBV, latent membrane protein 1 (LMP1), latent membrane protein-2A (LMP2A) and EBV-encoded nuclear antigen-1 (EBNA1), are involved in cHL pathogenesis. LMP1 mimics CD40 activation, LMP2A allows B cell development without B cell receptor (BCR) signaling and EBNA1 sustains EBV infection [24,25]. LMP1 increases the expression of discoidin domain receptor 1 (DDR1), a collagen receptor involved in HRS cell survival and the production of cytokine/chemokines involved in TME formation, such as CCL5 [24,25,26]. EBNA1 can increase CCL20, a chemokine involved in regulatory T cell (Treg) recruitment by HRS cells [27]. The TME of EBV+cHL patients is characterized by the presence of cytotoxic T lymphocytes against EBV-infected HRS cells and a high amount of histiocytes, dendritic cells and endothelial cells [24], suggesting that EBV+HRS cells use different mechanisms to escape antitumor immune responses [24,25]. Recently, novel treatments targeting EBV+cHL cells were developed, including vaccination with EBV proteins or peptides, gene therapy, the reactivation of EBV into the lytic cycle to kill infected cells, the antiviral drug ganciclovir and EBNA1 inhibitors [27,28].

Although cHL is not considered an AIDS-defining cancer, in the HIV-infected patients the risk of developing cHL is higher than in the general population [25,29]. EBV is present in almost all HIV-related cHL cases, and plays an important role in the pathogenesis of this disease [25,29]. The cooperation of these viruses, leading to immunodeficiency and chronic inflammation, creates the conditions for the development of cHL. Due to the moderate immune suppression resulting from the combination antiretroviral therapy (cART), the HIV-associated TME is characterized by a significant reduction in the number of HRS-associated CD4+ lymphocytes, and a decrease in functional and mature NK cells, dendritic cells and B cells, whereas CD8+ lymphocytes are maintained. Interestingly, the HIV virions released from activated CD4+ lymphocytes presented the survival factor CD40L [25,29]. Rosetting T cells are often replaced by spindle-shaped CD163+ macrophages [25,30] in the HIV-associated TME. 

The development of multiplexed technologies [1] provided the rationale for treatment approaches to specifically target HRS cells and/or to counteract the immunosuppressive functions of HRS cells and the TME. In this context, drugs such as brentuximab vedotin (BV), an anti CD30 antibody linked to the microtubule disrupter monomethyl auristatin E (MMAE), nivolumab or pembrolizumab (anti PD-1 antibodies) [31], and avelumab (anti-PD-L1 antibody) [32] have entered in clinical practice. Moreover, the Chimeric antigen receptor T cell (CAR T) therapy, which consists of the reinfusion of autologous T cells genetically engineered to target specific antigens expressed by HRS cells (CD30, LMP-1/2, CD123) and by the TME (CD19 and CD123), is also under testing in current clinical trials [33,34,35]. 

Several in vitro and in vivo preclinical studies discovered new drugs or new drug combinations able to target HRS cells and the TME, or reduce TAM infiltration in tumor tissues [36,37,38]. However, the efficacy of cancer therapy is limited by the occurrence of resistance that can be intrinsic, acquired (due to drug exposure) or TME mediated. The TME-mediated drug resistance can be ascribed to the secretion by noncancerous cells of soluble factors and EVs, or to their direct contact with HRS cells [39]. As a consequence, new drugs or drug combinations that show great efficacy in vitro and in tumor xenograft have not always demonstrated potential in clinic. For example, researchers found that BV resistance, obtained by continuous exposure of HRS to BV, was related to multidrug resistance protein 1 (MDR1) induction [38] and these preclinical studies, were translated into clinical studies. A phase I clinical trial of BV in combination with the MDR1 inhibitors cyclosporine (CsA) or verapamil hydrochloride was performed in patients with relapsed or refractory cHL [38]. Treatment of BV-resistant patients with BV and CsA resulted in better but not complete responses, suggesting that enhanced MDR1 activity is not the only mechanism involved in BV resistance. Consistently, Bankov et al. [15] demonstrated that HRS cells in direct contact with fibroblasts were protected against the cytotoxic effects of BV. Thus, the lack of a complete response with the combination of BV and MDR1 inhibitors in BV-resistant patients could be attributed, at least in part, to fibroblasts of the TME [38]. Moreover, BV resistance could be related to many other factors, such as intrinsic resistance to MMAE or to antibody–drug conjugates [40], increased NF-kB activation [41], CD30 shedding by metalloproteinases expressed by tumor cells or by other cells of the TME [14].

Given the complex role of TME in cHL progression, it is of fundamental importance to discover the most important microenvironmental interactions involved in the formation of an immunosuppressive TME and in resistance to anticancer therapy; evaluate the prevalence or proportion of certain cell subtypes; reveal the expression of molecules that can predict drug activity in order to modulate anticancer therapy and to avoid pointless toxicity. To this end, new techniques were developed to study the cellular composition of the TME and especially the cHL niche, the spatial disposition of noncancerous cells surrounding HRS cells and the expression of immunosuppressive molecules in relation to diagnosis, prognosis, and drug toxicity. Furthermore, new in vitro methods capable of mimicking the direct and indirect interactions with the TME were developed in order to evaluate drug activity and to discover molecules and mechanisms involved in the protection against anticancer drugs and in the building of an immunosuppressive and drug-protective TME. In vivo models of cHL (tumor xenografts) were used to evaluate the effects of anticancer drugs on tumor growth and TME composition. Genome-wide analyses, such as the sequencing of micro-dissected HRS cells and circulating tumor DNA, have led to more refined molecular characterization of HRS cells [3]. Proteomic studies were performed to identify biomarkers of primary refractory disease [42]. 

**Figure 2:**
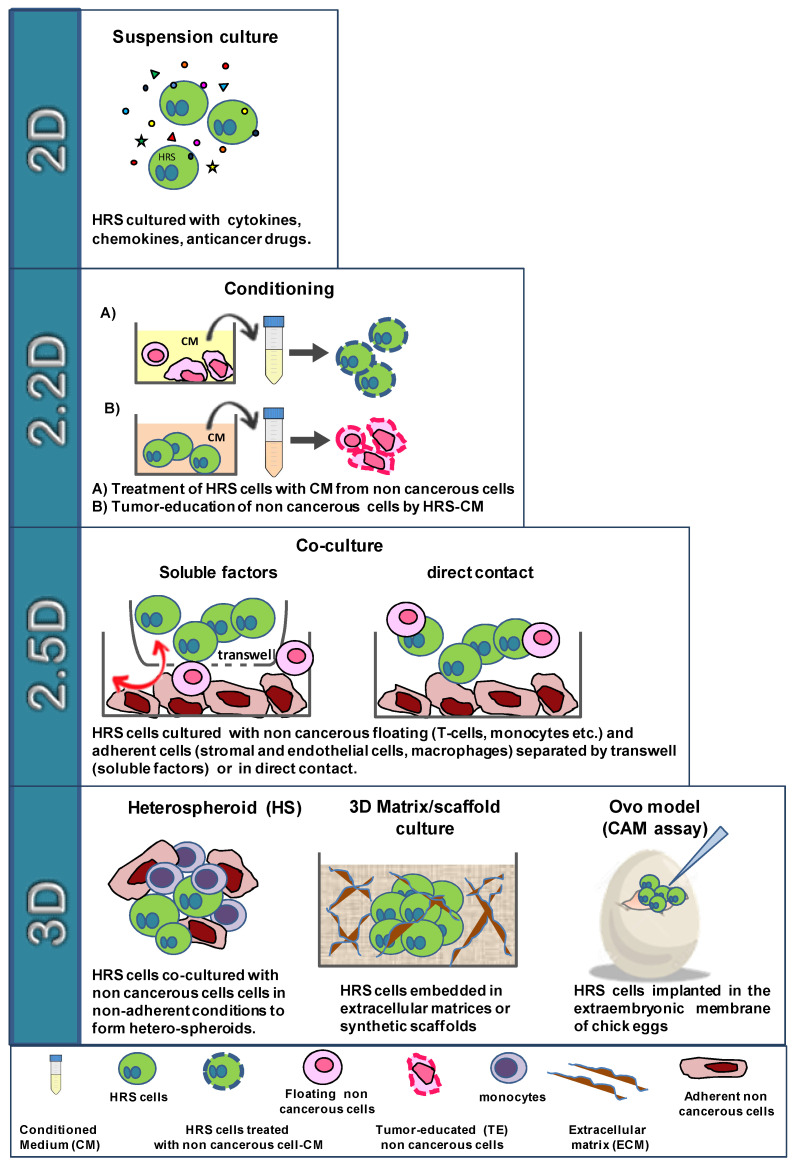
Schematic representation of the classification by complexity of the in vitro culture models used to study. The interactions of HRSs with the noncancerous cells of the tumor microenvironment.

Here, we describe the current knowledge regarding current and new methods adopted to study the TME composition and phenotype, functional studies used to clarify the interactions of HRS cells with the TME and to evaluate drug activity, results obtained with the most recent techniques and the present and future applications of these new techniques and their integration. Finally, we propose a classification of in vitro culture methods with growing complexity, used to study TME interactions in cHL: 2 dimensional (2D), 2.2D, 2.5D, and 3D models (Figure 2).

## 2. Characterization of the TME Composition

The analysis of HRS cells can reveal novel biomarkers and molecular regulators involved in tumor growth, metastasis and drug resistance, but this information should be integrated with the study of the cross-talk of tumor cells with the TME [43]. 

The use of immuno-oncology agents has emerged as an effective approach in the fight against cancer, especially in cHL; thus, it is fundamental to understand the immune escape mechanisms working in each patient. To achieve this, we need a precise characterization of the noncancerous cells that comprise the TME and their spatial relationships with respect to HRS cells. These studies should identify markers of drug resistance and response to therapy in cHL patients and promote the realization of personalized therapies.

Much information has remained largely inaccessible due to the limitations of currently established tools and methods. However, in the past few years, different new multiplexed methodologies allowing the simultaneous visualization of several antigens in the same specimen have emerged, improving clinical analysis and translational research.

Given that currently established techniques allow anatomy pathologists to examine a few protein markers and to evaluate their expression by visual examination, several more complex methods were recently developed to study the TME: the multiplex colorimetric immunohistochemistry (mIHC) [44] and the multiplex immunofluorescence (mIF) [45,46] associated with the spatial distribution of different cell populations using quantitative spatial analysis (qSA) (qmIF)[47], the cyclic immunofluorescence (CycIF) [48], the MultiOmyx™[49], the Multiplexed Ion Beam Imaging (MIBI) (bandura) [50,51], codetection by indexing (CODEX) [52] and digital spatial profiling (DSP) [53,54]. Digital image analysis (DIA) is used to transform and analyze imagines on a computer [55]. An overview of tissue imaging methods and a detailed description of multiplex techniques used to study the TME of different types of lymphoma were recently described [55,56]. These new techniques can provide important and efficient means to apply in clinic for prognostic evaluations and therapy selection, as well as for translational research, but they certainly require the collaboration of a multidisciplinary team, including pathologists, oncologists, immunologists and biologists. 

### Identifying the Immunoprotective cHL Niche with Multiplex Platforms

**Figure 3:**
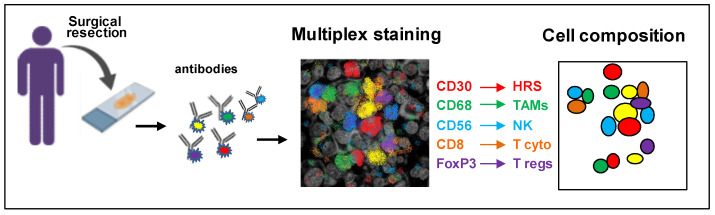
Schematic representation of multiplex immunohistochemistry. Different antibodies conjugated with different fluorophores can simultaneously detect different cell types and their spatial relationship in formalin-fixed, paraffin embedded sections of a reactive lymph node.

The development of multiplexed techniques for the simultaneous identification of multiple biological markers on a single tissue sample increases the opportunity to understand the interactions of HRS cells with the TME. These powerful strategies can allow important information, including: a more precise identification of specific cell types and individual cell phenotypes; the identification of the spatial relationship between multiple cell types; the co-localization of phenotypic and activation markers on individual cell types to define their functional states; and the preservation of tissues due to the simultaneous evaluation of multiple markers using a single tissue section [56] (Figure 3).

Roemer et al. [57] determined the nature and prognostic significance of the alterations of the immune checkpoint pathway PD-L1 and PD-L2 in cHL. Biopsy from patients with newly diagnosed cHL was used to evaluate the genetic alterations of PD-L1 and PD-L2. Dual-chromogenic IHC with PD-L1/-L2 and PAX5 demonstrated that HRS cells express PD-L1 and that the amplification at 9p24.1 of the PD-L1 and PD-L2 genes was more frequent in patients with advanced stage disease and shorter progression free survival (PFS) [57]. With the same technique, Roemer et al. [58] found that the reduced expression of the major histocompatibility complex (MHC) class II on HRS cells was associated with a favorable outcome, while decreased MHC class I, with inferior outcome independently of 9p24.1 status.

**Figure 4:**
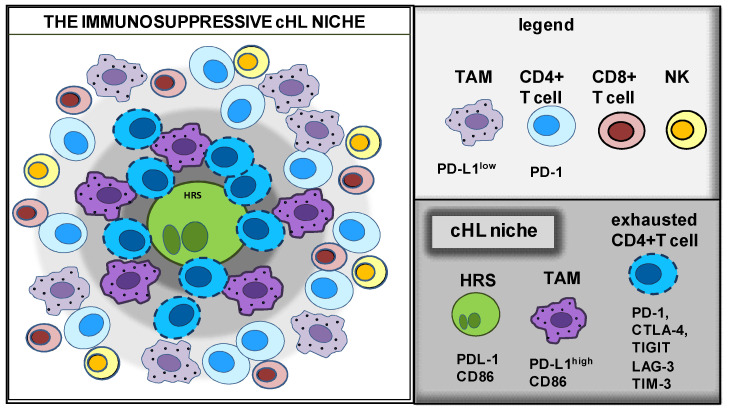
Schematic representation of the immune suppressive cHL niche. Cell composition and molecules expressed by different cell types of the cHL niche.

Using qmIF, Carey et al. [20] identified a novel micro-anatomic structure in cHL tumor biopsy, the “immunoprotective cHL niche” (Figure 4). They discovered that HRS cells reside within an enriched population of PD-L1^+^ TAMs in contact with “exhausted” PD-1^+^ T-cells. In the immune-protective niche, PD-L1^+^ TAMs that are close to PDL-1^+^ HRS cells can engage PD-1 on T-cells and help tumor cells to counteract immune-mediated killing. 

To verify the presence of correlations among the presence of myeloid-derived suppressor cells (MDSCs), TAMs and T-regs in cHL tissues, Au et al. [59] studied their distribution in cHL-TME. MDSCs can be subdivided into granulocytic MDSCs (G-MDSCs) and monocytic MDSCs (M-MDSCs). Using the MultiOmyx^TM^ immunofluorescence technique and 13 surface markers, high levels of both M-MDSCs and G-MDSCs were found in cHL tissues, with a higher frequency of G-MDSCs with respect to M-MDSCs. The levels of T cytotoxic cells correlated positively with M-MDSCs. Tregs were closer to M2-TAMs than to MDSCs and both Tregs and M2 TAMs were closer to G-MDSCs than to M-MDSCs. 

T cell immunoreceptor with Ig and ITIM domain (TIGIT) regulates T cell function, inhibits NK function and the secretion of proinflammatory cytokines and seems a promising target for cancer immunotherapy [60]. Li et al. [61] evaluated the expression of TIGIT in the T cell background surrounding HRS cells and histiocytic cells. A microenvironment tissue microarray was created from formalin-fixed normal human tonsil and tissue samples of HL-lymph nodes. Multiplex immunohistochemistry was used to evaluate the expression levels of TIGIT, PD-1, and of the lymphocyte markers CD3, CD8, CD4, FOXP3. TIGIT colocalized with PD-1; however, its expression in T cells was highly variable between cHL patients.

More recently, to obtain a 3D reconstruction of the complex niche of HRS cells, Annibali et al. [62] employed immunohistochemistry on serial sectioning and confocal microscopy. Using a new TIGIT scoring system, they found that TIGIT is expressed in CD4^+^ T lymphocytes surrounding HRS cells and there is a mutually exclusive expression of TIGIT and PD-L1. HRS cells surrounded by TIGIT-negative T lymphocytes were usually PD-L1⁺ while PD-L1^−^ HRS cells were surrounded by TIGIT^+^ lymphocytes. The authors suggested that HRS cells could use, as an alternative system to the PD1/PD-L1 interactions, the immune escape mediated by TIGIT.

Patel et al. [7], using multiplex immunofluorescence microscopy with digital image analysis, demonstrated that cHL is highly enriched for T cells expressing the cytotoxic T lymphocyte-associated protein 4 (CTLA-4). Rosetting T cells are less frequently positive for PD-1 or LAG-3 than for CTLA-4. The ligand of CTLA-4, CD86, is expressed by HRS cells and a subset of TAMs. CTLA-4⁺ T cells and CD86⁺ TAMs, are highly enriched in proximity of HRS cells. CTLA-4⁺ T cells in contact with HRS cells were found in recurrent cHL tumors after different therapies, including PD-1 blockade. The authors suggested that patients with cHL refractory to PD-1 blockade could benefit from CTLA-4 blockade [7]. El Halaby et al. [63] discovered that the immune-checkpoint regulators LAG-3 and TIM-3 are almost always present in the cHL TME. 

Using multiplex immunohistochemistry with tyramide signal amplification (TSA), Karihtala et al. [64] demonstrated that high proportions of PD-L1⁺ and IDO-1^+^ TAMs are both associated with unfavorable outcomes in cHL patients treated with standard chemotherapy. Conversely, the levels of PD-L1^+^ tumor cells, or TAM-negative for PD-L1 and IDO were not associated with a bad outcome, suggesting that the immunophenotype of TAMs, rather than their quantity, can affect the survival of cHL patients.

Roussel et al. [65] found that mass cytometry analysis defined distinct immune profile in B cell lymphomas, including Hodgkin lymphoma. They showed that canonical macrophage markers CD163 or CD68 cannot be used solely to define TAMs from lymphomas, and that S100A9, CCR2, CD36, Slan and CD32 should be included in future studies. Jachimowicz et al. [66], using whole-slide image analysis of the TME, identified low B cell content as a predictor of adverse outcome in patients with advanced-stage cHL treated with BEACOPP. 

The glycoprotein CD47 is often overexpressed on the surface of tumor cells and is usually considered a bad prognostic factor and a novel immunotherapeutic target [67]. CD47 is a strong ‘do not eat me’ signal that helps cancer cells to escape the recognition and the destruction by macrophages expressing signal regulatory protein alpha (SIRPα). Immunohistochemistry (tissue microarray) was used to evaluate CD47 in HRS cells from cHL tissues [68,69]. CD47 overexpression in HRS cells is frequent and there are no significant differences between cHL patients with high and low expression respect to the clinical stage, histological subtype sex, EBV status or the presence of B symptoms. cHL patients with high CD47 expression on tumor cells have an inferior event-free survival (EFS) compared with patients with low expression [69]. The blocking of the CD47–SIRPα axis checkpoint is currently under investigation in clinical trials for the treatment of relapsed or refractory hematologic malignancies, including cHL [70].

Overall, these new techniques, in conjunction with traditional histopathological techniques, represent a powerful tool to elucidate how HRS cells survive in the milieu of potentially hostile immune cells and to discover their immune evasive strategies involving the immune checkpoint proteins PD-L1, CTLA-4, LAG-3, TIM3, IDO-1, PD1, TIGIT and SIRPα, as well as other non-checkpoint mechanisms. Most important, many of these immune checkpoint molecules, such as PD-L1-PD1, TIGIT, CD80/CD86-CTLA-4, IDO-1 and CD47–SIRPα, are targetable and provide potential therapeutics to treat refractory and relapsed cases. Moreover, these studies revealed that to enhance disease diagnosis and prognosis, the sole characterization of tumor cells, in terms of expressed and secreted molecules, is not sufficient, as the composition of the TME is essential to depict the entire scenario. Actually, not only the cell number of TAMs, T cells, or CAFs forming the TME is fundamental, but also their spatial disposition and especially their phenotype. 

## 3. Identify the State of the Disease with Gene Expression Profiling of the TME

Gene expression profiling (GEP) can simultaneously measure the expression levels of thousands of genes and was applied to the study of the TME components using tissue biopsies [71]. GEP analysis was also used to find gene expression signatures associated with treatment outcomes or risk stratification at diagnosis in cHL [72,73,74]. Luminari et al. [72] used GEP to predict the response to ABVD. GEP was performed using biopsies from patients before and after treatment with chemotherapy. Results suggested its use to anticipate the early metabolic response (interim-FDG-PET) to therapy. Johnston et al. [73] used GEP to analyze the TME in biopsies from children diagnosed with cHL and found that that TME is prognostic for treatment effects in patients with intermediate risk. Jachimowicz et al. [74] demonstrated the association of three genes, TNFRSF8 (encoding CD30), CCL17 (encoding TARC) and PDGFRA, with progression-free survival (PFS) in advanced-stage BEACOPP-treated cHL patients and suggested the GEP assay for risk assessment before treatment.

## 4. In Vitro Models to Study HRS/TME Interactions

Functional studies, integrated with the study of the TME composition, the spatial organization and the molecules expressed by the different cell types, attempt to clarify the mechanisms leading to tumor progression and drug resistance that are mediated by the cross-talk of HRS cells with the TME. 

2D cell cultures are the standard for many different applications, including cancer research or regenerative medicine. Hence, almost all of our knowledge about fundamental biological processes has been provided by healthy cells, primary and established cell lines cultured as 2D adherent monolayer (solid tumors, the healthy counterparty stromal and epithelial cells) or as floating cells (hematological malignancies, healthy counterparty white blood cells and platelets). However, to study the direct interaction of tumor cells with the TME, we need techniques that best represent their cross-talk, including 3D models and heterospheroids (HSs). 

Given the high complexity of TME interactions, we propose a classification with four different methods, characterized by increased complexity, used recently and in the past to perform functional in vitro studies with HRS cells: 2D, 2.2D, 2.5D, 3D (Figure 2). 

In 2D, HRS cells are cultured in suspension culture in the presence of molecules such as cytokines/chemokines, anticancer drugs, etc. In 2.2D, HRS and noncancerous cells are cultured with their respective products (conditioned medium or EVs) to study their mutual conditioning in a paracrine manner. In the 2.5D co-culture method, HRS cells are cultured together with noncancerous cells (floating or adherent cells) in direct contact or separated by a Transwell insert. In the 3D, which more closely resembles the in vivo network, HRS cells are embedded in extracellular matrix (ECM) scaffolds, implanted in developing chick eggs and cultured with noncancerous cells under non-adherent conditions to form heterospheroids (HSs), in ECM scaffolds or sponge scaffolds (Figure 2). 

The first step (2D model) to study TME interactions is the cultivation of HRS cells (free floating cells) with antitumor agents or molecules known to be secreted by the TME, such as recombinant cytokines. The effects are evaluated directly on HRS tumor cells and usually include tumor growth, migration, apoptosis, drug resistance, autophagy, immunosuppressive activity (such as the expression or secretion of immune suppressive molecules, like PDL-1, IL-10, IL-13) or modulation of key molecules (such as CD30, needed for BV efficacy), etc. (Figure 2).

The 2.2D method is used to mimic the paracrine HRS/TME cross-talk mediated by their respective products, such as conditioned medium or EVs, without any direct cell–cell contact. One main example of a 2.2D method is the “tumor education” of noncancerous cells by HRS cells, which consists of the treatment of these cells (monocytes, stromal cells, T-cells, endothelial cells etc.) with conditioned medium from tumor cells. Noncancerous cells become protumorigenic and are then analyzed and compared with untreated cells for their phenotype, secretion of cytokines/chemokines and functional activities. In turn, the conditioned medium of tumor-educated noncancerous cells is used on HRS cells to evaluate its tumor-promoting effects, including drug resistance and immunosuppressive activity (Figure 2).

The 2.5D method evaluates the consequences of the co-cultivation of HRS with TME cells that are cultured in direct contact or in Transwell inserts. In the Transwell culture system, different cell types are separated in two compartments by a porous membrane that only permits the continuous exchange of soluble factors. This method is used to study the short-distance interplay among different cell types. Usually, in 2.5D, HRS cells are co-cultured with T lymphocytes, monocytes, and eosinophils or over a layer of adherent stromal cells, endothelial cells, or macrophages. 

### 4.1. The Cross-Talk of HRS Cells with T lymphocytes

The most abundant population in the cHL-TME is T cells, which comprise T helper cells, regulatory T cells, and cytotoxic T cells [75,76]. Various molecules are involved in the interactions between HRS cells and the surrounding T cells including the adhesion molecules CD54, CD58 expressed by HRS cells and CD11a/CD18 (LFA-1); CD2 expressed by T cells; the co-stimulatory molecules CD40, CD80, CD86 on HRS cells and their ligands CD40L and CD28 on the rosetting T cells; and the suppressing molecule PD-L1 on HRS cells and PD-1 on T-cells [77]. 

The consequences of CD40 engagement on HRS cells was evaluated with soluble CD40L (2D) or with CD40L+ Jurkat T cells (CD4+ T cell line expressing CD40L) coculture (2.5D) [22]. CD40 engagement on HRS cells increased both tumor growth and survival, upregulated CD54, CD80, and IRF4 expression, augmented the secretion of IL-8, TNF-α, IL-6, LT-α and CCL5, decreased Fas-mediated apoptosis and increased NF-kB activation [4]. In turn, by secreting CCL5 and CCL17/TARC, HRS cells recruited T lymphocytes and then educated them to become exhausted/anergic T cells that supported tumor cell survival [19]. HRS cells directly recruited the immunosuppressive Tregs, [19] or recruited and then educated CD4^+^ T cells to differentiate into CD25^+^ Foxp3^+^ Tregs, thus promoting their accumulation in the cHL niche [78,79,80,81].

One main immune evasion mechanism of HRS cells is mediated by their expression of PD-L1 [58] which engages PD-1 expressed by T cells leading to T cell “exhaustion”, i.e., the progressive loss of T cell effector function and antitumor immune response. Blockade of the PD-1/PD-L1 pathway rescues T cells from the inhibitory effects exerted by HRS cells and restores a T cell-mediated antitumor immune response [19]. 

Jalali et al. [82] found another function of PD-L1 in HRS cells. They demonstrated that PD-L1 engagement, by both membrane-bound and soluble PD-1, induced reverse signaling in HRS cells that, through the activation of the MAPK pathway, enhanced HRS cell proliferation and decreased apoptosis. PD-1 blockade counteracted this signaling, suggesting an additional mechanism for the clinical responses driven by the anti-PD-1 antibodies immune checkpoint inhibitors in cHL.

Wein et al. [23] discovered that T helper cells from cHL lymph nodes are polarized towards a Treg phenotype by comparing the gene expression profile of CD4^+^ T cells from cHL lymph nodes with the profiles of T cells from reactive tonsils. This evidence was further confirmed by the co-cultivation of HRS cells with CD4^+^ T cells and CD25-depleted CD4^+^ T cells from healthy donors, which demonstrated HRSs capability to hijack T helper cells to become Tregs. However, the authors discovered that the molecules involved in the polarization of T helper cells towards Tregs (IL-4, IL-6, IL-15 and PG-E2) were expressed not only by HRS cells but also by the TME [23], suggesting that tumor-educated noncancerous cells, such as TAMs or CAFs, can help tumor cells to build the immunosuppressive TME [19]. When performing co-culture systems with T cells, the effect of HLA mismatching has to be considered. Since the cultivation of HRS cells together with allogenic CD4^+^ T cells is capable of inducing a potent antitumoral activity that became detectable after about 6 days [83], to avoid this effect, co-culture experiments were performed for only 72 h [23]. The co-cultivation of HRS cells with T cells was also used to demonstrate that the binding of CD200^+^ HRS cells to the inhibitory CD200R and BTLA receptors, expressed by cHL-infiltrating T cells, can affect T cell function and represent an additional mechanisms of immune escape [23] and that the expression of high levels of extracellular adenosine (eADO), likely due to the downregulation of adenosine deaminase in both HRS and Tregs, can modify T-cell function [23].

Recently, to study the interactions between HRS cells and rosetting T cells, Veldman et al. [5] used a short-term in vitro coculture model. To avoid alloreactivity, they cultured tumor cells with peripheral blood mononuclear cells (PBMCs) matched to the HLA-II type of HL cell lines. After co-cultivation, cytospin specimens were prepared to search for T-cell rosettes (HRSs surrounded by T cells). The antigen-dependent communication between T cells and HRS cells, defined as immunological synapse, was studied combining immunofluorescence with in situ proximity ligation assay [84], a technique used to observe protein–protein interactions related to their cellular localization. The authors identified the physical interactions between CD2-CD58 and CD4-HLA-II, with CD2 and CD4 expressed by T cells, and CD58 and HLA-II expressed by HRS cells. These findings revealed that the interactions of CD4-HLA-II, and especially of CD2-CD58 are involved in the activation of HRS cells by rosetting T cells [5].

Zocchi et al. [85] used the co-cultivation of HRS cells or HL-MSCs with CD8^+^ T lymphocytes to investigate the role of lymph node MSCs in the modulation of tumor cell recognition by effector T lymphocytes. They found that the overexpression of ADAM10 by HRS cells, together with increased release of NKG2D ligand (NKG2D-L), reduced T cell cytolytic activity. NKG2D is an activating receptor expressed on NK cells, which play a pivotal role in tumor immunosurveillance. Once NKG2D is engaged by its ligand, NKG2D-L, NK and γδT lymphocytes initiate a rapid immune response against tumor cells. ADAM10 is a metalloproteinase that mediate the proteolytic shedding of surface molecules. Thus, HRS cells, by secreting NKG2D,-L may cause the internalization and downregulation of NKG2D receptor on effector NK cells, which nullifies their antitumor response capabilities.

### 4.2. The Cross-Talk of HRS Cells with Eosinophils

The presence of a prominent tissue eosinophilia [77] represents a typical histopathology hallmark of Hodgkin disease. In vitro studies demonstrated that HRS cells, by secreting CCL5, can recruit eosinophils [81] and mast cells, [86] the predominant CD30L-expressing cells in HL-affected lymph nodes [86]. Eosinophils may act as important elements in the pathology of HL by providing cellular ligands for CD40 and CD30. CD40L and CD30L expressed by eosinophils are functionally active and able to transduce proliferative signals on CD40^+^ and CD30^+^ HRS cells [87,88], suggesting that they may contribute, likely together with other cell types, to CD30 and CD40 activation of tumor cells in cHL lymph nodes.

### 4.3. The Cross-Talk of HRS Cells with Monocytes 

TAMs can subvert local immune surveillance by expressing cell surface proteins or by releasing soluble factors that exert immunosuppressive functions [89,90]. TAMs are generally classified in two subtypes with opposite functional activity, the classical activated M1-TAMs and alternatively activated M2-TAMs. M1-TAM exerts antitumor functions, while M2-TAM can promote cancer progression by inhibiting the antitumor immune response and promoting metastasis and angiogenesis. These two TAM subtypes are characterized by different cytokine/chemokine secretions and different cell surface molecules expression [89,90]. 

To study the tumor education of monocytes by molecules secreted by HRS cells, researchers used human monocytes purified from PBMC and/or the human monocytic cell line THP-1 [91].

Conditioned medium from HRS cells (HRS-CM) increased the migration of monocytes [80] and “educated” monocytes to become pro-tumorigenic M2-TAMs [18,19,36,37,80,92,93], characterized by the expression of high levels of CD206, IDO, and PD-L1 and by the secretion of CCL17/TARC, TGF-β, and IL-10. In turn, conditioned medium from HRS-educated monocytes inhibited the proliferation of PHA-activated lymphocytes and increased the clonogenic growth of HRS cells [37,80]. A semi-solid culture system (3D) (methyl cellulose) was also used to demonstrate that monocytes migrate towards and came into direct contact with HRS cells [94].

Arlt et al. [18] found that CD206 expression in HRS-educated monocytes was higher than in monocytes cultured with M-CSF and that this phenomenon was mainly regulated by IL-13. CD206 is involved in collagen degradation through a mannose receptor–mediated pathway [95]. The microarray analysis of cHL tissues revealed an high expression of CD206 in patients with stage IV disease, indicating that the presence of M2-TAM with high CD206 expression and likely with expected increased matrix-remodeling capacity, could characterize advanced stages of cHL [18].

The notion that high levels of immunosuppressive TAMs are a bad prognostic factor [9,96] in cHL, prompted the use of novel therapeutic strategies aimed to target both HRS cells and TAMs. In this context, recent in vitro and in vivo studies demonstrated that both trabectedin [37] and the PI3Kd/Ƴ inhibitor RP6530 [36] target both HRS cells and, directly or indirectly, monocytes. Casagrande et al. [37] found that trabectedin killed HRS cells and, by decreasing the secretion of immunosuppressive cytokines/chemokines, reduced the capability of HRS-CM to recruit monocytes and to hijack them towards an immune-suppressive M2-TAM phenotype [37]. Locatelli et al. [36] demonstrated that RP6530, beside killing HRS cells, downregulated lactic acid metabolism, switching the activation of macrophages by HRS-CM from an immunosuppressive M2-like phenotype to a more inflammatory M1-like state.

### 4.4. The Cross-Talk of HRS Cells with Fibroblasts and MSCs

The majority of cells, with the exception of hematopoietic cell lineages and few others, are anchorage dependent. The co-cultivation of HRSs with cells that grow in adherent conditions forming 2D monolayers (fibroblasts, MSCs, and endothelial cells) are usually performed using Transwell systems (coculture without contact) or culturing HRS cells directly on the cell layer. These co-culture methods are referred to “2.5D” [97]. Alternatively, conditioned medium or EVs can be utilized for a paracrine interaction (2.2D method). In particular, the 2.2D is applied to evaluate the effect of specific cytokines and the protective effects of stromal cells against anticancer drugs. However, even if it is quite easy to study the effects of soluble molecules, the risk is the loss of important information, such as drug effects on noncancerous cells or the cooperation mediated by the direct contact of HRSs with noncancerous cells, including the protective effects and the tumor education. In contrast, HRS cultured directly on a stromal cell layers showed a strong adherence which made separation for subsequent molecular analysis of the individual populations very difficult, unless purifying HRS cells by cell sorting or using anti-CD30 beads [80].

To study the cross-talk HRS/MSCs (2.5D), researchers employed the immortalized HS-5 fibroblast-like cell line, commercial MSCs derived from bone marrow, adipose tissues, and MSCs purified from cHL tissues (HL-MSCs).

Meadows et al. [98] proved that co-cultivation of HRS cells with a layer of HS-5 cells (2.5D model) increased CCL5 secretion. This effect was decreased by the PI3Kd inhibitor GS-1101, able to block Akt activation. 

By secreting tumor-promoting factors, HRS cells and HL-MSCs mutually influence their growth, movement, and anticancer drug sensitivity [15,80,99]. HRS cells, by secreting CCL5, can recruit HL-MSCs, which in turn can recruit monocytes [80] and protect tumor cells against drug activity [15]. Conditioned medium from HL-MSCs (HL-MSCs-CM) can decrease Auranofin gold(I) cytotoxic activity in HRS cells [100] and IL-7 secreted by HL-MSCs can decrease doxorubicin effects [101]. In addition, the direct contact of HRS cells with fibroblasts or HL-MSCs, which are usually more effective than the derived CM, can protect against the anticancer activity of the proteasome inhibitor bortezomib [102] and of BV [15]. Conversely, the cytotoxicity of the NF-KB inhibitor DHMEQ is only slightly decreased by the direct contact of HRSs with HL-MSCs [103].

Zocchi et al. demonstrated that HL-MSCs, by secreting TGF-β, are able to downregulate NKG2D expression on effector T cells [85] and by expressing ADAM10 to decrease NKG2D-L in tumor cells, thus reducing the anticancer immune response [85].

## 5. Extracellular Vesicles in HRS-TME Communication and Immune Suppression

Extracellular vesicles (EVs) are lipid-bound vesicles secreted by cells into the extracellular space and are found in many body fluids. EVs play a fundamental role in cell communication transporting to local and distant sites biologically active molecules. EVs can contain and carry diverse materials, such as lipids, proteins, RNA, glycolipids and metabolites [104]. EVs allow the interactions with the near-TME, but especially with distant sites. EVs secreted by tumor cells can modify receiver cells and favor tumor progression, immune suppression, angiogenesis and metastasis formation in different cancer models [104]. EVs can carry lipophilic and hydrophilic drugs, representing an additional manner for anticancer drug delivery. Thus, EVs are considered a potential target for novel therapeutic strategies or a vehicle for anticancer drugs [105].

Dorsam et al. [99] reported that HRS cells secrete EVs (HRS-EVs) able to educate fibroblasts to became CAFs. cHL vesicles obtained from HRS-CM are internalized by fibroblasts, which increase their migratory capacity, growth (G-CSF- and GM-CSF-dependent) and the release of pro-inflammatory cytokines (e.g., IL-1α, IL-6, and TNF-α) and pro-angiogenic factors (VEGF). In HRS-tumor xenografts, the co-injection of KM-H2 cells with HRS-EVs, by reprogramming fibroblasts in CAFs and by increasing angiogenesis, leads to the formation of a tumor-promoting TME [99].

Tosetti et al. [106] demonstrated that ADAM10 is released in exosome-like vesicles (ExoV) by HRS cells and by HL-MSC. The cross-talk mediated by ExoV (ADAM10+) between HL-MSCs and HRS cells determined the release of soluble CD30 (sCD30), mechanism that could interfere with the responses to BV. ADAM10 inhibitors, LT4 and CAM29, by counteracting the ADAM10 sheddase activity performed by ExoV and the consequent release of sCD30, maintained/restored the cytotoxic effects of BV [106]. Thus, ADAM10 inhibitors could counteract the formation of the immunosuppressive TME [107] and improve BV anticancer activity [106].

Hansen et al. [108] discovered that HRS cells release CD30 on EVs. These EVs stimulate IL-8 secretion by granulocytes in a CD30-dependent manner. Drees et al. [109] found that tumor-associated miRNAs levels in plasma EVs are predictive of metabolic tumor activity in cHL patients.

Small extracellular vesicles (SEVs) secreted by HRS cells were detected in circulating plasma from pediatric Hodgkin lymphoma [110]. Circulating CD30+ SEVs were evaluated with the AuNP Aptasensor (aurum nanoparticles with peroxidase activity) to monitor the status of cHL patients [111].

In conclusion, the cargo function of EVs secreted by HRS cells (ADAM10, TNF-α, sCD30) may modify the TME composition by educating noncancerous cells to support HRS cells, decrease the anticancer activity of BV, and EVs may be used to evaluate drug response and patient status [105]. Thus, a deeper investigation of EVs could improve our knowledge about TME interactions, allowing the discovery of mechanisms involved in drug resistance or to monitor the status of cHL patients.

## 6. Trogocytosis in the Formation of the Immunosuppressive TME

Trogocytosis is the process in which fragments of cell membranes are transferred from one cell to another [112]. In vitro co-cultivation studies demonstrated that PDL-1, CD137, CD30 and CD83 are transferred by trogocytosis from HRS cells to noncancerous cells, suggesting this process as an additional mechanism involved in the building of the immunosuppressive TME [113].

Kawashima et al. [94] proved that HRS cells can transfer PD-L1/L2 by trogocytosis to monocytes. Although the induction of PD-L1 by HRS-CM needs at least 3 days [19], the transfer by trogocytosis is a rapid phenomenon and requires the proximity of HRS cells to monocytes. Trogocytosis was observed via confocal microscopy after 1 h incubation of HRS cells stained with anti-PD-L1 and monocytes labeled with the lipophilic fluorescent dye PKH67. The co-localization of PD-L1 with PKH67, visualized by confocal microscopy, demonstrated the direct transfer of the molecule from HRS cells to monocytes. After a short incubation time, PD-L1 was upregulated only in TAMs in close contact with HRS cells and not in monocytes co-cultured with HRS cells with low/absent expression of PD-L1. Thus, the transfer of PDL-1 by trogocytosis is another mechanism to evade antitumor immunity in the cHL niche (Figure 4) and likely in cHL-HIV patients where monocytes substitute rosetting T-cells [25]. 

HRS cells express CD137 that can be transferred via trogocytosis to the neighboring HRSs and antigen-presenting cells (APC) that express the CD137L. Then, the CD137-CD137L complexes are internalized, leading to the loss of CD137L and, as a consequence, to the reduced costimulation of T cells through CD137 [114,115,116]. CD137 engagement can induce the secretion of the immunosuppressive cytokine IL-13 by HRS cells [114].

Nakashima et al.[117] found that CD30 expressed by HRS cells can extract CD30L from the adjacent cell in a trogocytic manner, resulting in actin-mediated internalization of the complex. 

Li et al. [118] demonstrated that CD83 is a promising target for cHL treatment. HRS cells express CD83 and secrete sCD83 that inhibited T cell proliferation. CD83 can be also transferred from HRS cells to T cells by trogocytosis, leading to the upregulation of PD-1 in the trogocytosed (CD83^+^)CD4^+^ T cells that may become further suppressed by PD-L1 engagement.

## 7. 3D Models to Study Tumor–Stroma Interactions and Drug Activity

The cHL-TME, composed of different cell types and an abundant ECM, can promote tumor growth, invasion, the malignant phenotypes, and protect against anticancer therapy [77]. In different cancer types [119,120,121,122,123], including cHL [37,124,125], cells in 3D cultures show significant differences in terms of morphology, gene/protein expression profile, growth rate, invasive capability, and sensitivity to anticancer drugs with respect to 2D cultures. 

Since the 2D in vitro models only partially represent the complex and dynamic interactions of the TME and ignore spatial cell–ECM and cell–cell interactions, new in vitro 3D models were developed [119,120]. These new 3D models, recently developed to study the HRS/TME cross-talk and drug activity, could bridge the gap between oversimplified 2D systems and, at least in part, the unrepresentative animal models that frequently fail to recapitulate human cancer progression and to reproduce adverse effects (Figure 2).

**Figure 5:**
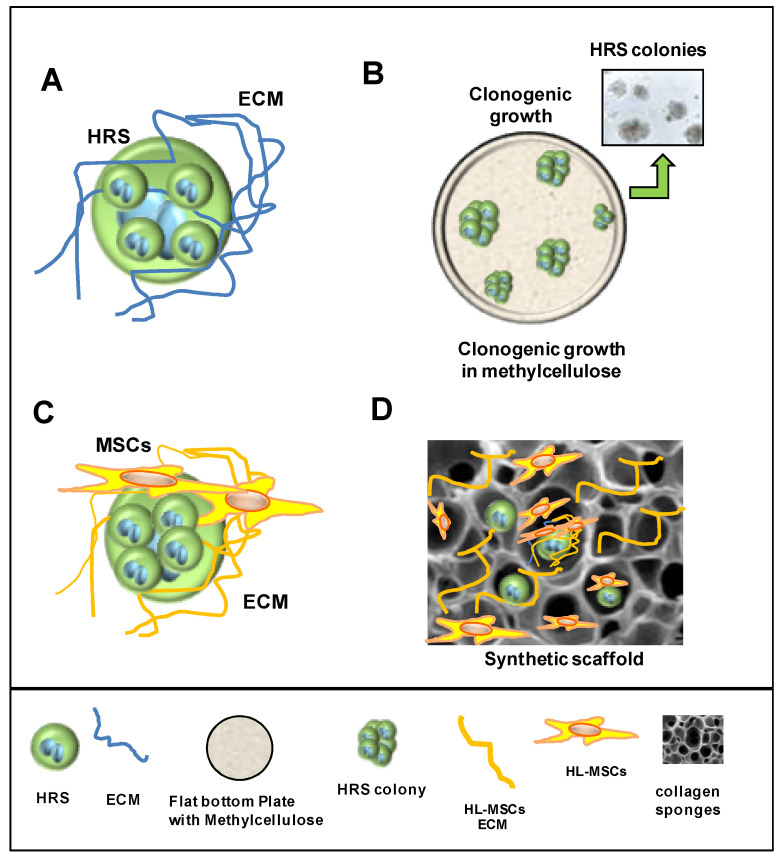
3D models used to study cHL/TME interactions and drug activity: human and synthetic scaffolds. (**A**) HRS cells are cultured with soluble collagen or embedded in scaffolds constituting hydrogels that can be derived from animals (Matrigel^®^, collagen), plants (alginate/agarose), or synthesized from chemicals (QGel^®^ Matrix, methylcellulose). (**B**) Methylcellulose assay. HRS cells are cultured in methylcellulose medium to form colonies. (**C**) Decellularized extracellular matrices (ECM scaffolds) derived from patient lymph nodes are repopulated with HL-MSCs followed by the addition of HRS cells. (**D**) Commercial sponges made of microfibrillar collagen (Avitene^TM^ Sponges) are used as synthetic scaffold. Sponges were repopulated with HL-MSCs, which produce the ECM, and then with HRS cells. Both the ECM scaffolds and the repopulated synthetic scaffold can be used to evaluate drug activity.

**Figure 6:**
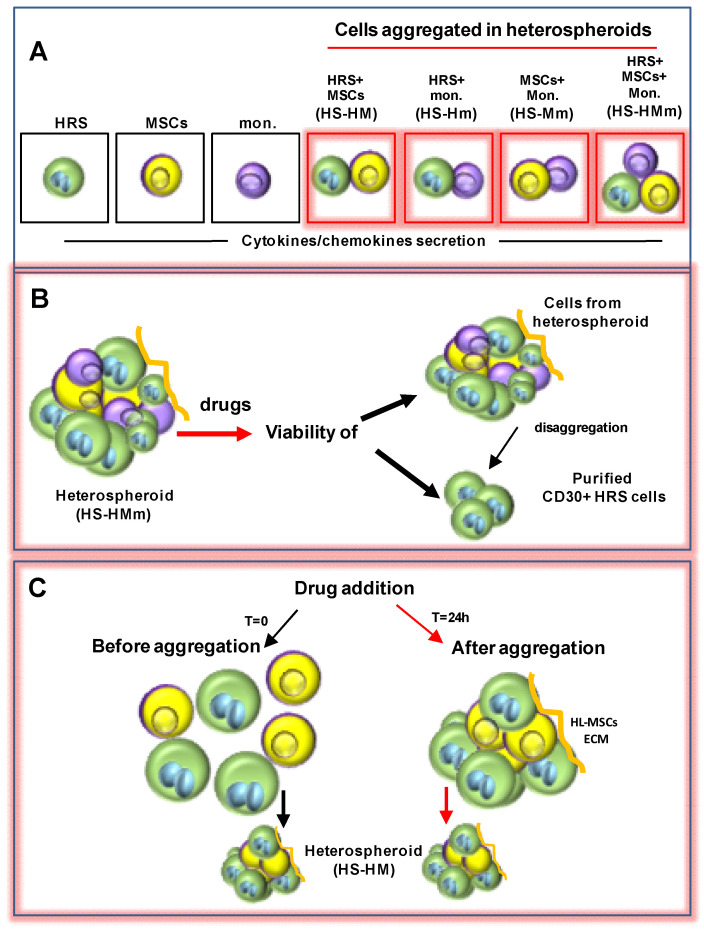
3D models used to study TME effects on cytokine secretion and drug activity. To obtain the spontaneous aggregation in the 3D heterospheroids (HS), HRS cells, HL-mesenchymal stromal cells (HL-MSCs) and monocytes (mon.) are cultured in non-adherent conditions (poly-HEMA coated wells). (**A**) Cytokine secretion is evaluated in the different cell type combinations. (**B**) Heterospheroids (HS-HMm) are used to evaluate the cytotoxic effects of single drugs or drug combinations. After treatment, viability is evaluated in cells from HS and in purified CD30^+^HRS cells. (**C**) To determine if the direct contact with HL-MSCs can induce protective effects, drugs are added before (T = 0) and after (T = 24h) the aggregation of HRS cells and HL-MSCs in HS-HM.

**Figure 7:**
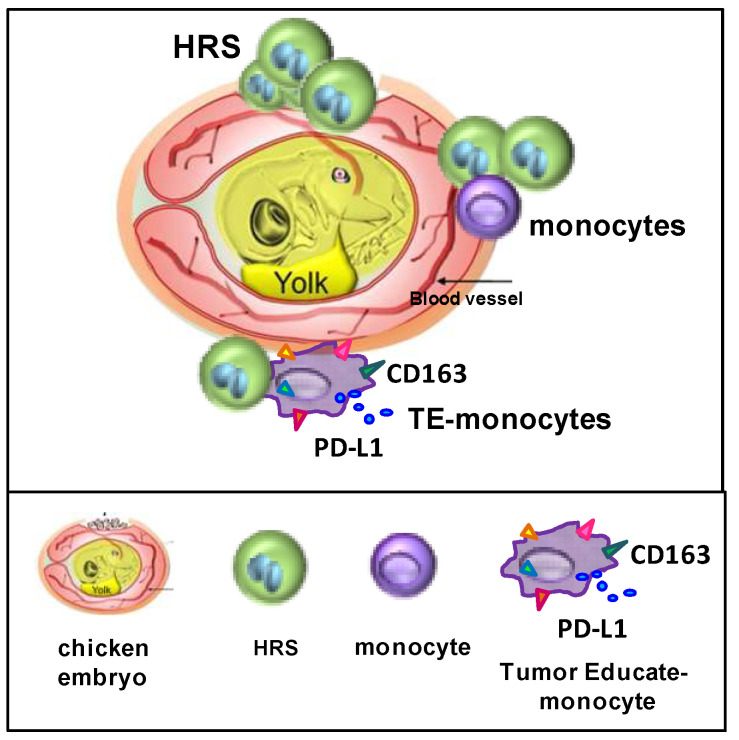
Chick chorioallantoic membrane (CAM) model. HRS cells are implanted alone, with monocytes or tumor-educated monocytes in the extra–embryonic membrane of developing chick eggs.

To mimic the HRS/TME interactions, different techniques were used: cultivation of HRS cells in 3D-matrix [125] (Figure 5A); cultivation of HRS cells in methylcellulose medium (Figure 5B) [126]; cultivation of HRS with HL-MSCs in human or synthetic scaffolds [124] (Figure 5C,D); cultivation of HRS cells under non-adherent conditions with HL-MSCs with and without monocytes, to form multicellular heterospheroids (HS) (Figure 6) [37,80,124,127]; and implantation of HRS cells alone [17] or with monocytes [18] on the extraembryonic membrane of developing chick eggs (chick chorioallantoic membrane, CAM assay) (Figure 7). Then, we describe the 3D models listed by complexity.

### 7.1. Cultivation in 3D Extracellular Matrix

The TME of cHL, and especially the most frequent nodular sclerosis (NSCHL) subtype, contains a large number of fibroblasts and a collagen-rich ECM. To mimic the 3D architecture formed by fibrillar ECM proteins, HRS cells were cultured in scaffolds constituting of hydrogels or in inert matrices (Figure 5). Hydrogels are polymer materials with hydrophilic properties derived from animals (collagen, Matrigel), plants (alginate/agarose), or chemical synthesized (methylcellulose, QGel^®^ Matrix). Inert matrices are sponge-like membranes made of polystyrene that contain pores allowing the proliferation and growth of cells. HRS and HL-MSCs were also cultured in scaffolds constituting of the ECM derived from HL-MSCs (Figure 5C) or in inert matrices [124] (Figure 5D).

Birgersdotter et al. [125] compared the gene expression profile of cHL-derived L-1236 cells cultured in suspension culture (2D) or embedded in collagen (3D) (Figure 5A) to that of laser-microdissected HRS cells recovered from cHL tumor tissues. The authors investigated if the cultivation of the L-1236 cell line in a 3D matrix could render L-1236 cells more similar to HRS cells recovered from cHL tissues. They found that the cultivation of L-1236 in the 3D matrix affected the gene expression of L-1236 cells, inducing a more tumor-related expression profile, characterized by the upregulation of genes involved in immune response and apoptosis, and the downregulation of genes involved in cell division [125].

The metalloproteinase ADAM10 mediates the release of CD30 by HRS cells. CD30 is released as a cleaved soluble ectodomain (soluble CD30, sCD30) and as an intact molecule embedded in the membrane of EVs. Hansen et al. [128], using HRS cells embedded in growth factor-reduced Matrigel, proved that CD30^+^ EVs secreted by HRS cells were retained in the matrix, whereas sCD30 penetrated rapidly into the surrounding culture medium. Even if CD30 shedding by ADAM10 causes a gradual depletion of CD30 binding sites for BV in HRS cells, the adherence of CD30-EVs, but not of sCD30, to CD30L^+^ mast cells could allow the binding of BV to the CD30L+ cells of the TME [128]. 

Semi-liquid methylcellulose medium was used to evaluate the clonogenic growth of HRS cells (Figure 5B) in the presence of stimuli-like growth factors [129], conditioned medium from noncancerous cells [80] and anticancer drugs [37,100]. Using this simple technique, it is possible to evaluates the capability of HRS cell to form a colonies, thus identifying only the proliferating fraction of cells and their self-renewing ability [126]. 

Linke et al. [17] demonstrated the positive effects of HRS cells on human umbilical vein endothelial cells (HUVEC) sprouting using HUVEC spheroids generated by their mixing with methylcellulose for 24h. The formed HUVEC spheroids were seeded with Matrigel mixed with KM-H2 conditioned medium (KM-H2-CM) and photographs of each spheroid were taken to evaluate the sprouting frequency of endothelial cells. 

Kawashima et al. [94] cultured in 50% methyl cellulose PKH67-labeled monocytes and HRS-mOrange^+^ cells. Using confocal microscopy, they showed that monocytes migrate towards and come into direct contact with HRS cells. 

### 7.2. Heterospheroids (HS) to Study the Interactions of HRS Cells with TME and Drug Activity

To mimic the interactions of stromal cells with tumor cells, 3D HSs formed by HRS cells and HL-MSC (HS-HM) or HRS cells, HL-MSC, and monocytes (HS-HMm) cultured under non-adherent conditions, were developed [37,80,124,127] (Figure 6A–C). To prevent cell attachment, plates were coated with poly-HEMA (hydrophilic polymer) allowing cells to cluster together. Under these experimental conditions, cells at time 0 were dispersed in the medium and after 24 h they started to self-assemble, forming 3D HS [80]. Cells grow in the absence of a supporting scaffold and produce their own ECM and spatial organization. These in vitro models were developed to mimic the TME interactions leading to tumor progression and, in particular, the immunosuppressive tumor education of noncancerous cells and the protection against anticancer therapy [130].

#### 7.2.1. Heterospheroids (HS) to Study ADAM10 Inhibitors in Combination with BV

Pece et al. [124] used HS-HM-heterospheroids, obtained by the cultivation of HRS cells with HL-MSCs under non-adherent conditions (Figure 6) or in collagen scaffolds (Figure 5C), to evaluate if ADAM10 inhibitors (LT4 and MN8), could increase the anti-lymphoma effects of BV by decreasing CD30 shedding. They found that in HS-HM, the ADAM10 inhibitors LT4 and MN8: reduced ATP content and glucose consumption (cell proliferation); increased lactate dehydrogenase (LDH), (cell damage); reduced HS dimension; decreased the shedding of both CD30 and TNFα; increased BV activity. 

The same authors, to more closely recapitulate the architecture of cHL, used decellularized extracellular matrices (ECM scaffolds) derived from patient lymph nodes repopulated with HL-MSCs (Figure 5C) and HRS, to evaluate the ADAM10 inhibitory activity. They found that LT4 and MN8 reduced CD30 shedding and growth of the CD30^+^HRS cells in this experimental condition. However, since this latter 3D system was difficult to standardize in terms of scaffold size, shape and structure, commercial sponges made of microfibrillar collagen (Avitene^TM^ sponges) were later used instead of ECM. Sponges were repopulated with HL-MSCs and then with HRS cells (Figure 5D). The effects observed in HS-HM were similar in ECM scaffolds and sponge scaffolds repopulated with HL-lymph node-derived matrix. ADAM10 inhibitors capable of enhancing BV activity in HRS cells cultured over a layer of HL-MSCs (2.5D) and in HS-HM (HS-non adherent conditions) exerted additive effects in repopulated scaffolds [124].

#### 7.2.2. Heterospheroids (HS) to Study the TME-Protective Effects against Drugs

Heterospheroids (HS-HMm) formed by HRS cells, HL-MSCs and monocytes cultured under non-adherent conditions, were used to evaluate CCL5 secretion (Figure 6A) and to study drug activity [80]. The cultivation of HRS cells with HL-MSCs (HS-HM) enhanced CCL5 secretion, which was further increased by the addition of monocytes (HS-HMm) (Figure 6A) [80]. The co-cultivation of HRS cells with monocytes (HS-Hm) did not modify CCL5 secretion.

Heterospheroids were also used to evaluate the anticancer activity of the CCR5-antagonist maraviroc in cHL. Maraviroc counteracted the aggregation of CCR5^+^ HRS cells with HL-MSCs and monocytes in heterospheroids (HS-HMm) and decreased the number of viable cells and colonies formed by HRS cells [80]. To evaluate maraviroc activity specifically on CD30^+^ HRSs, tumor cells recovered from HS-HMm after trypsinization, were purified with anti-CD30 beads (Figure 6B). Heterospheroids (HS-HMm) were also used to perform drug combination studies, showing that maraviroc was capable of enhancing doxorubicin activity [80]. 

Heterospheroids formed by HRS cells and HL-MSCs (HS-HM) were used to perform drug combination studies with maraviroc and trabectedin, a potent anticancer drug capable of reducing the viability of HRS cells in a 2D model and in HS-HM heterospheroids [37]. Maraviroc and trabectedin, used as single agents, decreased the viability of the formed HS-HM in a dose-dependent manner, and their combination further increased this effect [127]. To demonstrate that the direct contact of HRS cells with HL-MSCs could reduce drug cytotoxic effects, maraviroc and trabectedin were added before (t = 0) and after the spontaneous cell aggregation in HS (T = 24h) (Figure 6C). Cell viability was evaluated after 6 days of incubation with the drugs. The results showed that maraviroc and trabectedin used alone reduced cell viability, but their combination was significantly more efficacious than the single treatments. These effects were more evident when drugs were added before cell aggregation, demonstrating the protective role of the direct contact of HRS cells with MSCs [127].

## 8. The CAM Model to Study Dissemination and Interactions of HRSs with Monocytes

HRS cells with metastatic potential exit the tumor lymph node via efferent lymph vessels or through high endothelial venules and migrate/disseminate to the next functional node [131]. Celegato et al. [100] found that HRS-CM (VEGF+) increased the tubulogenesis (2D model) of human umbilical vein endothelial cells (HUVECs). In addition, Linke et al. [17] studied the interactions of HRS cells with endothelial cells and demonstrated that HRS cells induced endothelial cell migration and angiogenesis by secreting VEGF. Fhu et al. [132] reported that lymphotoxin-α (LTα) secreted by HRS cells acts on endothelial cells to upregulate the expression of adhesion molecules involved in T cell recruitment.

The CAM assay is a well-established in vivo model used to study angiogenesis, wound healing, tumor growth, and metastasis formation [133]. This assay consists of the implantation of cells, materials or compounds on the extraembryonic membrane of developing chick eggs (Figure 7). The CAM assay (ovo model) was used to evaluate the proangiogenic potential of HRS cells by Linke et al. [17,134]. With this 3D in vivo model, they demonstrated that the autocrine activation of WNT signaling in HRS cells promoted both motility and tumor growth, and increased HRS-mediated angiogenesis. As a consequence, WNT might function as a positive regulator of lymphoma dissemination by affecting cHL cell chemotaxis and also by increasing angiogenesis. Consistently, in cHL patients, high levels of *VEGFA* was associated with poorer overall survival [17] (Figure 7).

Arlt et al. [18] used the CAM assay (ovo model) to study the interaction of HRS cells with monocytes and tumor-educated monocytes (TE-monocytes). HRS cells (L-428, L-1236) mixed with TE-monocytes were embedded in Matrigel and applied onto the CAM. After four days, the tumors were excised, fixed, embedded in paraffin or Tissue Tek and characterized macroscopically and histologically. The tumor volume and the topography of lymphoma cells and monocytes in relation to lymphatic and blood vessels were evaluated. These preclinical studies revealed that cultivation of HRS cells with TE-monocytes in CAM supports the dissemination of HRS cells via lymphatic vessels, even if tumor size and vessel destruction were decreased in comparison with lymphoma cells implanted alone (Figure 7). 

## 9. In Vivo Tumor Xenograft to Study TME Interactions 

The potential to find drugs with dual effects, i.e., the capability to target tumor cells and modify the TME, were further evaluated using the cHL tumor xenograft. Indeed, after drug treatment, it is possible not only to assess tumor growth but also the composition of tumor tissues, including the number and type of TAMs, CAFs and to monitor angiogenesis. 

Thanks to in vivo studies with HRS xenografts, the dual mechanism of action of auranofin [100], trabectedin [37], maraviroc [80], and the dual PI3Kd/g inhibitor RP6530 [36] were demonstrated. These drugs inhibited tumor growth, reduced the number of TAMs, determined M2-TAM repolarization into proinflammatory macrophages, and decreased angiogenesis in HRS xenografts. In cHL patients, RP6530 showed objective responses in a phase I trial that were associated with a significant inhibition of circulating MDSCs and a reduction in serum levels of the poor prognosis factor CCL17/TARC [36].

In vivo studies were also performed by Dörsam et al. [99] to confirm HRS-EVs activity in fibroblasts. HRS cells and fibroblasts were subcutaneously injected into the lower flank of NOD scid gamma (NSG) mice. The mice were then treated with intravenous injection of HL-EVs over time and the control animals with only PBS. Tumor tissues from mice treated with HL-EVs were characterized by higher vascularization and fibroblasts with a CAF phenotype. The conclusion was that HRS-EVs could promote a suitable TME for tumor growth and progression. 

The CD123/IL-3 receptor is expressed by both HRS cells and TAMs [93,129]. Ruella et al. [93] demonstrated that CART123 cell therapy targeting HRS cells as well as the M2-TAMs of the immunosuppressive TME caused the complete and long-lasting remission of HL tumor xenografts and established immunological memory. 

## 10. Conclusions

Novel approaches used to study the composition of the cHL-TME have contributed to the discovery of new therapeutic strategies, new prognostic markers and the understanding that not only the number of noncancerous cells but also their spatial disposition (formation of a protective niche) together with their phenotype and functional characteristics, determine the fate of HRS cells. Moreover, the combination of different techniques allowed the discovery of different mechanisms involved in the building of the immunosuppressive and protective TME. HRSs can communicate and hijack the TME using soluble factors, EVs and direct cell–cell contact, through which they can transfer immunosuppressive molecules to adjacent cells (trogocytosis).

Finally, the use of new 3D models to mimic the cHL niche, allowing the cultivation of HRS with different cell types, may help to discover new mechanisms involved in TME formation, drug resistance and to evaluate new drugs or drug combinations capable not only of killing cancer cells but also of disrupting the protective cHL niche.
